# Apolipoprotein E mRNA expression in mononuclear cells from normolipidemic and hypercholesterolemic individuals treated with atorvastatin

**DOI:** 10.1186/1476-511X-10-206

**Published:** 2011-11-10

**Authors:** Alvaro Cerda, Fabiana DV Genvigir, Maria AV Willrich, Simone S Arazi, Marcia MS Bernik, Egidio L Dorea, Marcelo C Bertolami, Andre A Faludi, Mario H Hirata, Rosario DC Hirata

**Affiliations:** 1 Department of Clinical and Toxicological Analyses, School of Pharmaceutical Sciences, University of Sao Paulo, Sao Paulo, Brazil; 2 University Hospital, University of Sao Paulo, Sao Paulo, Brazil; 3 Dante Pazzanese Institute of Cardiology, Sao Paulo, Brazil

**Keywords:** apolipoproteina E, hypercholesterolemia, single nucleotide polymorphism (SNP), APOE gene expression, atorvastatin

## Abstract

**Background:**

Apolipoprotein E (apoE) is a key component of the lipid metabolism. Polymorphisms at the apoE gene (*APOE*) have been associated with cardiovascular disease, lipid levels and lipid-lowering response to statins. We evaluated the effects on *APOE *expression of hypercholesterolemia, *APOE *ε2/ε3/ε4 genotypes and atorvastatin treatment in Brazilian individuals. The relationship of *APOE *genotypes and plasma lipids and atorvastatin response was also tested in this population.

**Methods:**

*APOE *ε2/ε3/ε4 and plasma lipids were evaluated in 181 normolipidemic (NL) and 181 hypercholesterolemic (HC) subjects. HC individuals with indication for lowering-cholesterol treatment (n = 141) were treated with atorvastatin (10 mg/day/4-weeks). *APOE *genotypes and *APOE *mRNA in peripheral blood mononuclear cells (PBMC) were analyzed by TaqMan real time PCR.

**Results:**

HC had lower *APOE *expression than NL group (p < 0.05) and individuals with low *APOE *expression showed higher plasma total and LDL cholesterol and apoB, as well as higher apoAI (p < 0.05). Individuals carrying ε2 allele have reduced risk for hypercholesterolemia (OR: 0.27, 95% I.C.: 0.08-0.85, p < 0.05) and NL ε2 carriers had lower total and LDL cholesterol and apoB levels, and higher HDL cholesterol than non-carriers (p < 0.05). *APOE *genotypes did not affect *APOE *expression and atorvastatin response. Atorvastatin treatment do not modify *APOE *expression, however those individuals without LDL cholesterol goal achievement after atorvastatin treatment according to the IV Brazilian Guidelines for Dyslipidemia and Atherosclerosis Prevention had lower *APOE *expression than patients with desirable response after the treatment (p < 0.05).

**Conclusions:**

*APOE *expression in PBMC is modulated by hypercholesterolemia and the *APOE *mRNA level regulates the plasma lipid profile. Moreover the expression profile is not modulated neither by atorvastatin nor *APOE *genotypes. In our population, *APOE *ε2 allele confers protection against hypercholesterolemia and a less atherogenic lipid profile. Moreover, low *APOE *expression after treatment of patients with poor response suggests a possible role of *APOE *level in atorvastatin response.

## Background

Dyslipidemia is an important risk factor in the development of atherosclerosis and cardiovascular events. Research on cardiovascular diseases has leaded to a better knowledge of the molecular basis of atherosclerosis and the identification of a key role of apolipoprotein (apo) E in this process [1]. ApoE is a major constituent of triglyceride-rich chylomicrons, very low density lipoprotein (VLDL) and some subclasses of high density lipoprotein (HDL) particles, participating in the clearance of these particles from circulation by serving as a ligand for their catabolism via low density lipoprotein (LDL) and apoE receptors [2].

The gene encoding the apoE protein (*APOE*) is polymorphic resulting in three major isoforms (ε2, ε3 and ε4) caused by two single nucleotide polymorphisms (SNPs) in the exon 4 of the *APOE*, resulting in cysteine-arginine interchanges at residuals 112 and 158 of the protein, which has been associated with a number of pathophysiological conditions, including cardiovascular and neurological diseases [3].

The inhibitors of the 3-hydroxy-3-methylglutaryl coenzyme A reductase (HMGCR), or statins, are among the most prescribed drugs worldwide and provide extensive benefits in the prevention of primary and secondary cardiovascular diseases [4]. However, there is considerable interindividual variation in the lipid-lowering response to statins, which is attributed to the interaction between multiple environmental factors and genetic determinants involved in the pharmacokinetic and pharmacodynamic pathways of these drugs [5].

The *APOE *gene is among the most extensively studied genes involved in the statins pharmacodynamic. The studies investigating the potential modifying role of *APOE *genotypes on lipid response to statin therapy have produced data frequently contradictory and so far inconclusive [6]. Evidence suggests that allele ε4 carriers appear to have attenuated lipid-lowering response and ε2 carriers have enhanced response [5], however differential interpretation of these results among investigators difficult the consensus [6]. Moreover genome-wide association studies (GWAS) have also shown an association of the *APOE *locus with hypercholesterolemia but they were unsuccessful in establishing its relationship with statin response when whole-genome platforms were used [7].

The regulation of *APOE *expression is very complex with participation of several factors controlling its transcription, including some factors that also regulate the expression of other proteins that control the lipid traffic [1]. Nevertheless, possible regulation of *APOE *transcription by polymorphisms and statins has been poorly studied.

The purpose of this study is to investigate the effects on *APOE *mRNA expression profile of hypercholesterolemia and *APOE *genotypes using PBMC from normolipidemics and hypercholesterolemic individuals. Moreover, we also analyzed the influence of atorvastatin treatment on *APOE *expression in hypercholesterolemic individuals, as well as the relationship of *APOE *genotypes with plasma lipid and atorvastatin response in our population.

## Results

### Characteristics of study population

Main characteristics of normolipidemic and hypercholesterolemic individuals are shown in Table 1. Frequencies of ethnics, gender, family history of coronary artery disease (CAD), cigarette smoking and physical activity were similar between NL and HC groups. However, mean values of age and body mass index (BMI), as well as frequencies of menopause, hypertension and obesity were higher in HC than NL group (p < 0.05). As expected, HC individuals had a more atherogenic lipid profile showing higher total, LDL and VLDL cholesterol, triglycerides and ApoB compared with NL subjects (p < 0.05).

**Table 1:** Demographic and laboratory characteristics of study groups

Parameter	Hypercholesterolemic (n = 181)	Normolipidemic (n = 181)	p-value
Age, years	56.1 ± 10.8	46.9 ± 6.8	< 0.001
Ethnics [European/African], %	62/38	65/35	0.581
Gender, % of women	67	73	0.205
Menopause, %	52	19	< 0.001
Family history of CAD, %	55	45	0.062
Hypertension, %	57	36	< 0.001
Obesity, %	29	15	0.002
Cigarette smoking, %	16	18	0.673
Physical activity, %	49	45	0.553
BMI, kg/m^2^	27.8 ± 4.1	26.2 ± 4.2	< 0.001
Total cholesterol, mg/dL	270 ± 41	173 ± 18	< 0.001
LDL cholesterol, mg/dL	183 ± 36	98 ± 18	< 0.001
HDL cholesterol, mg/dL	57 ± 14	58 ± 12	0.386
Triglycerides, mg/dL	150 ± 65	82 ± 28	< 0.001
VLDL cholesterol, mg/dL	30 ± 13	16 ± 6	< 0.001
ApoAI, mg/dL	136 ± 26	141 ± 26	0.101
ApoB, mg/dL	143 ± 27	85 ± 23	< 0.001

Non-continuous variables are compared by chi-square test. Continuous variables are presented as media ± SD and compared by *t *test. CAD, coronary artery disease; LDL, low density lipoprotein; HDL, high density lipoprotein; VLDL, very low density lipoprotein; ApoAI, apolipoprotein AI; ApoB, apolipoprotein B. Conversion factors to convert to Systeme Internacional (SI) units are 0.02586 for cholesterol (mmol/l), 0.01129 for triglycerides (mmol/l) and 0.01 for apolipoproteins (g/l).

### Association of hypercholesterolemia and plasma lipids with *APOE *gene expression

**Figure 1:**
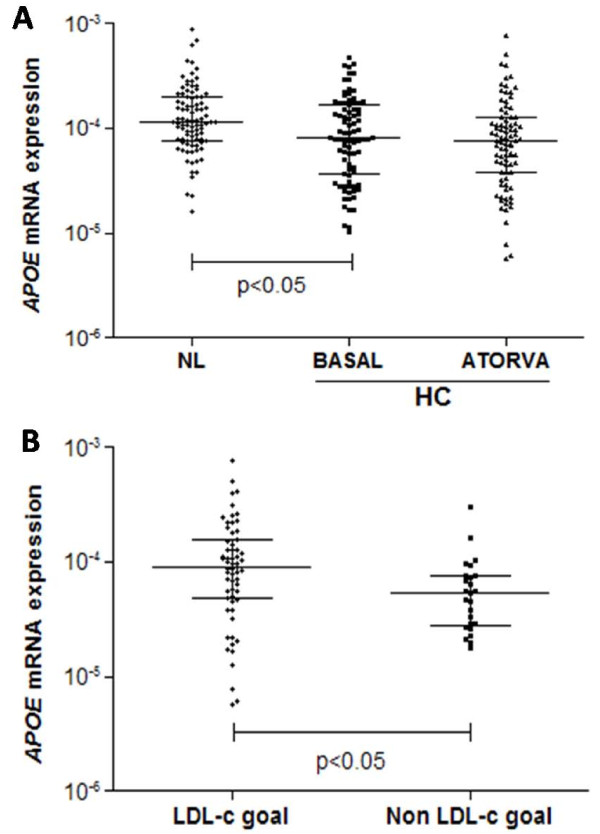
**PBMC *APOE *mRNA expression in normolipidemic (NL) and hipercolesterolemic individuals before (HC - BASAL) and after treatment with 10 mg/day of atorvastatin (HC- ATORVA) [A] and according to LDL cholesterol goal achievement in response to atorvastatin [B]**. Values are presented as dispersion graph with bars indicating median and interquartile range and compared by Mann-Whitney U test or Wilcoxon signed test.

As represented in the Figure 1A, NL individuals showed higher levels of *APOE *mRNA expression in PBMC than HC individuals at baseline (NL: median 11.7 × 10^-5 ^and inter-quartile (IQ) range: 7.6 × 10^-5 ^- 20 × 10^-5^; ATORVA baseline: median: 8.1 × 10^-5 ^and IQ range: 3.7 × 10^-5 ^- 17 × 10^-5^; p = 0.004).

Spearman's correlation test demonstrated that *APOE *mRNA expression in PBMC is negatively correlated with total cholesterol (r = -0.195, p = 0.008) and LDL cholesterol (r = -0.214, p = 0.004), and positively correlated with apoAI concentration (r = 0.202, p = 0.007) (data not shown).

**Figure 2:**
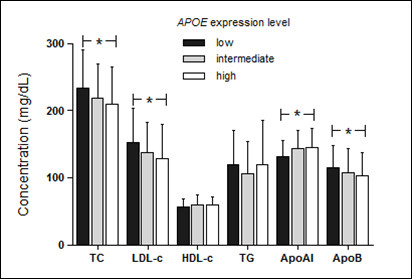
**Plasma lipid profile according to *APOE *mRNA expression levels**. Patients were grouped in levels of *APOE *mRNA expression according to tercile values (first tercile [low]: 2^-ΔCt ^< 7.7x10-5; second tercile [intermediate]: 2^-ΔCt^: 7.7 x10-5  - 17.1 x10-5; third tercile [high]: 2^-ΔCt ^> 17.1 x10-5) and plasma lipids were compared among the groups by one way ANOVA and Tukey test. TC, total cholesterol; LDL-c, low density lipoprotein cholesterol; HDL-c, high density lipoprotein cholesterol; TG, triglycerides; apoAI, apolipoprotein AI; apoB, apolipoprotein B; (*), p < 0.05. Conversion factors to convert to Systeme Internacional (SI) units are 0.02586 for cholesterol (mmol/l), 0.01129 for triglycerides (mmol/l) and 0.01 for apolipoproteins (g/l).

Due to the wake lineal correlation and in order to corroborate the association of *APOE *mRNA expression levels with plasma lipid concentration and hypercholesterolemia, comparison analyses by grouping patients according to tercile of *APOE *mRNA values were performed (first tercile: 2^-ΔCt ^< 7.7x10^-5^; second tercile: 2^-ΔCt^: 7.7x10^-5^ - 17.1x10^-5^; third tercile: 2^-ΔCt ^> 17.1x10^-5^). First, second and third tercile were considered as low, intermediate and high expression, respectively. As observed in Figure 2, individuals with low *APOE *mRNA expression had higher plasma concentration of total and LDL cholesterol and apoB and lower levels of apoAI (p < 0.05). Further univariate logistic regression analysis demonstrated that individuals with mRNA values into the low expression group had increased risk to hypercholesterolemia than individuals with higher (intermediate and high expression) values of *APOE *expression (OR: 2.06, 95% C.I.: 1.10-3.87, p = 0.025). Moreover, when compared with the individuals with the highest *APOE *mRNA levels, to belong to the low expression group represent an increased risk of 2.25 (OR: 2.25, 95% CI:1.08-4.67, p=0.030).

### Relationship of *APOE *genotypes with basal lipids, atorvastatin response and *APOE *expression

Genotype and allele frequencies are presented in Table 2. Frequencies of SNP genotypes were as expected from a HWE (p > 0.05, data not shown) in both, NL and HC groups. The genotype distribution was different between NL and HC groups (p = 0.003) showing higher frequencies of ε2ε3 and ε2ε4 genotypes in NL individuals. ε2 allele was more frequent in NL group (7.7%) than in HC (1.7%, p < 0.001) and logistic regression analysis showed that ε2 carriers had decreased risk of hypercholesterolemia (OR: 0.27; C.I.: 0.08 - 0.85; p < 0.025), after adjustment for covariates gender, ethnics, history of CAD, age, hypertension, obesity, cigarette smoking, physical activity and alcohol consumption.

**Table 2:** Genotype and allele frequencies  of *APOE *polymorphisms in hypercholesterolemic and normolipidemic individuals.

Genotypes	ε2ε2	ε2ε3	ε2ε4	ε3ε3	ε3ε4	ε4ε4
HC (n = 181)	0.0% (0)	2.8% (5)	0.6% (1)	66.3% (120)	26.5%(48)	3.8% (7)
NL (n = 181)	0.6% (1)	9.4% (17)	5.0% (9)	61.3% (111)	22.7%(41)	1.1% (2)
	χ^2 ^= 17.624; 5 *df*; p = 0.003					

**Alleles**	ε2	ε3	ε4			

HC	1.7%	80.9%	17.3%			
NL	7.7%	77.4%	14.9%			
	χ^2 ^= 15.223; 2 *df*; p < 0.001					
	
	Allele	O.R.	95%C.I.			p-value
	
	ε3	1.00	--			--
	ε2	0.27	0.08-0.85			0.025
	ε4	1.31	0.76-2.26			0.331

Number of individuals is in parenthesis. HC, hypercholesterolemic; NL, normolipidemic; *df*, degrees of freedom; O.R., odds ratio; C.I. confidence interval. O.R. values and 95% C.I. were obtained from logistic regression analysis using hypercholesterolemia as dependent variable with gender, ethnics, history of coronary artery disease, age, hypertension, obesity, cigarette smoking, physical activity and alcohol consumption as covariates for the model. Influence of ε2 and ε4 alleles on risk for hypercholesterolemia was accessed comparing with the reference allele ε3.

Individuals carrying the ε2 allele showed lower total and LDL cholesterol and apoB, as well as higher HDL cholesterol (p < 0.05) compared with the ε3 and ε4 carriers in NL group (Table 3). No differences were observed on basal plasma lipids in HC group according to *APOE *genotypes.

**Table 3:** Influence of *APOE *polymorphisms on basal serum lipids in normolipidemic and hipercholesterolemic individuals

Parameter	ε2	ε3	ε4	p-value
***Normolipidemics***	(18)	(111)	(43)	**--**
Total cholesterol, mg/dL	160 ± 23^a^	173 ± 18^b^	177 ± 15^b^	0.003
LDL cholesterol, mg/dL	81 ± 18^a^	99 ± 18^b^	105 ± 16^b^	< 0.001
HDL cholesterol, mg/dL	65 ± 11^a^	57 ± 12^b^	56 ± 12^b^	0.023
VLDL cholesterol, mg/dL	14 ± 5	17 ± 6	16 ± 5	0.179
Triglycerides, mg/dL	72 ± 25	84 ± 28	80 ± 26	0.179
ApoAI, mg/dL	150 ± 20	140 ± 25	137 ± 31	0.205
ApoB, mg/dL	71 ± 19^a^	86 ± 23^b^	90 ± 22^b^	0.011
***Hypercholesterolemics ***	(5)	(120)	(55)	--
Total cholesterol, mg/dL	253 ± 25	271 ± 43	267 ± 31	0.668
LDL cholesterol, mg/dL	166 ± 22	183 ± 39	181 ± 25	0.622
HDL cholesterol, mg/dL	62 ± 18	57 ± 14	56 ± 13	0.679
Triglycerides, mg/dL	133 ± 42	151 ± 71	148 ± 52	0.713
VLDL cholesterol, mg/dL	27 ± 8	30 ± 14	30 ± 10	0.713
ApoAI, mg/dL	140 ± 34	138 ± 29	132 ± 19	0.418
ApoB, mg/dL	128 ± 29	143 ± 28	143 ± 25	0.450

Number of individuals is in parenthesis. Values are expressed as media ± SD and compared by one way ANOVA. HC, hypercholesterolemic; NL, normolipidemic; LDL, low density lipoprotein; HDL, high density lipoprotein; VLDL, very low density lipoprotein; ApoAI, apolipoprotein AI; ApoB, apolipoprotein B. ε2 represent ε2ε2 and ε2ε3 carriers; ε3 symbolize ε3ε3 genotype and ε4 represent ε3ε4 and ε4ε4 individuals. ^a, b^, different letters at the same line represent statistical significance. Conversion factors to convert to Systeme Internacional (SI) units are 0.02586 for cholesterol (mmol/l), 0.01129 for triglycerides (mmol/l) and 0.01 for apolipoproteins (g/l).

Association between *APOE *genotypes and basal plasma lipids in NL and HC individual was further explored in a multiple linear regression analysis adjusted for covariates gender, age, ethnics, hypertension, obesity, family history of CAD, physical activity, cigarette smoking and alcohol consumption. In women group, menopause status did not modified *APOE *genotypes effects, therefore this variable was not included in the regression analysis. In NL group, the presence of ε2 allele was associated with a significant decrease of 11.6, 18.1 and 13.8 mg/dL in values of total cholesterol, LDL cholesterol and apoB, respectively (Table 4). Moreover, ε2 allele was also associated with an increment of 9 mg/dL of HDL cholesterol. On the other hand, presence of ε4 allele was related to an increment of 6.7, 8.8 and 9.0 of total cholesterol, LDL cholesterol and apoB, respectively (Table 4). Confirming the previous results, we did not found any association between *APOE *genotypes and basal plasma lipids in HC individuals in the multiple linear  regression model (data not shown).

**Table 4:** Influence of *APOE *genotypes on basal serum lipids in normolipidemic individuals by multiple linear regression analysis

Variable	ε2	p-value	ε3	p-value	ε4	p-value
	B (SE)		B (SE)		B (SE)	
Total cholesterol	-11.6 (5.3)	0.030	-1.0 (3.3)	0.753	6.7 (3.3)	0.047
LDL cholesterol	-18.1 (5.2)	0.001	-0.7 (3.4)	0.847	8.8 (3.4)	0.010
HDL cholesterol	9.0 (3.4)	0.009	-1.2 (2.2)	0.568	-1.8 (2.2)	0.399
VLDL cholestrol	-1.8 (1.5)	0.236	0.8 (0.9)	0.352	-0.3 (0.9)	0.799
ApoB	-13.8 (6.9)	0.046	-2.6 (4.2)	0.534	9.0 (4.5)	0.047
ApoAI	11.3 (7.6)	0.138	0.7 (4.5)	0.879	-5.4 (4.9)	0.269

B and (SE) represent regression coefficient and standard error and are expressed in mg/dL. B values were obtained from multiple linear regression analysis introducing gender, ethnics, family history of coronary artery disease, age, hypertension, obesity, cigarette smoking, physical activity and alcohol consumption as covariates. HDL, high density lipoprotein; LDL, low density lipoprotein; VLDL, very low density lipoprotein; apoB, apolipoprotein B; apoAI, apolipotrotein AI. Conversion factors to convert to Systeme Internacional (SI) units are 0.02586 for cholesterol (mmol/l), 0.01129 for triglycerides (mmol/l) and 0.01 for apolipoproteins (g/l).

No differences were observed on *APOE *mRNA expression according to *APOE *ε2/ε3/ε4 genotypes (p > 0.05, data not shown). Moreover the genotype distribution among *APOE *mRNA expression level groups was similar (χ^2^ = 0.695, 4 *df*, p = 0.952; data not shown).

Atorvastatin treatment reduced the plasma lipids with exception of apoAI in ATORVA group (Additional file 1). Results of CK and ALT demonstrated that there were no muscle or liver adverse reaction cases during the four-week atorvastin treatment (data not shown).

*APOE *genotypes did not influence basal and post-treatment plasma lipids in individuals treated with atorvastatin (Table 5). Further multiple linear regression analysis confirmed these results (data not shown). Moreover, 10 mg/day atorvastatin treatment did not modify the *APOE *mRNA expression (p > 0.05; Figure 1A). Despite the absence of atorvastatin effects on *APOE *mRNA expression, those individuals without LDL cholesterol goal achievement according to the IV Brazilian Guidelines for Dyslipidemia and Atherosclerosis Prevention [8] had lower expression (p = 0.009; Figure 1B) than patients with desirable response after the treatment.

**Table 5:** Influence of *APOE *genotypes on serum lipids in hypercholesterolemic individuals treated with atorvastatin (10 mg/day/4-weeks)

Parameter		ε2 (5)	ε3 (92)	ε4 (44)	p-value
Total cholesterol,	*Basal*	253 ± 25	284 ± 39	275 ± 27	0.100
mg/dL	*Treatment*	174 ± 28	198 ± 31	198 ± 27	0.285
	*Change, %*	-32 ± 6	-30 ± 10	-28 ± 8	0.332
LDL cholesterol,	*Basal*	166 ± 22	194 ± 37	188 ± 21	0.166
mg/dL	*Treatment*	96 ± 20	116 ± 28	119 ± 22	0.262
	*Change, %*	-42 ± 9	-40 ± 13	-37 ± 10	0.408
HDL cholesterol,	*Basal*	62 ± 18	57 ± 15	55 ± 11	0.534
mg/dL	*Treatment*	55 ± 14	55 ± 14	53 ± 10	0.745
	*Change, %*	-11 ± 11	-3 ± 11	-3 ± 9	0.270
VLDL cholesterol,	*Basal*	27 ± 8	32 ± 14	31 ± 11	0.672
mg/dL	*Treatment*	24 ± 12	26 ± 12	26 ± 9	0.880
	*Change, %*	-10 ± 38	-14 ± 30	-12 ± 25	0.902
Triglycerides,	*Basal*	133 ± 42	159 ± 72	155 ± 53	0.672
mg/dL	*Treatment*	118 ± 58	130 ± 58	131 ± 43	0.880
	*Change, %*	-10 ± 38	-14 ± 30	-12 ± 25	0.902
ApoAI,	*Basal*	140 ± 34	138 ± 29	132 ± 19	0.418
mg/dL	*Treatment*	151 ± 54	140 ± 28	133 ± 24	0.259
	*Change, %*	+6 ± 16	+2 ± 11	+1 ± 13	0.650
ApoB,	*Basal*	128 ± 29	143 ± 28	143 ± 25	0.532
mg/dL	*Treatment*	97 ± 23	99 ± 21	97 ± 19	0.984
	*Change, %*	-24 ± 7	-30 ± 13	-31 ± 11	0.494

Number of individuals is in parenthesis. -/+ symbols in change indicate reduction or increment of each parameter. Values are presented as media ± SD and compared by one way ANOVA. LDL, low density lipoprotein; HDL, high density lipoprotein; VLDL, very low density lipoprotein. ε2 : ε2ε2 and ε2ε3 genotypes; ε3: ε3ε3 genotype; ε4: ε3ε4 and ε4ε4 genotypes. Conversion factors to convert to Systeme Internacional (SI) units are 0.02586 for cholesterol (mmol/l), 0.01129 for triglycerides (mmol/l) and 0.01 for apolipoproteins (g/l).

## Discussion

Diverse studies have proposed an important role of the *APOE *in hypercholesterolemia and statin response based in this association with *APOE *ε2/ε3/ε4 genotypes, but little information is known about the relationship of these variables with the expression status of *APOE*. Here, we describe the mRNA expression profile in PBMC from NL individuals and HC patients treated with atorvastatin.

Absence or structural mutations of *APOE *cause significant disorders in lipid metabolism and cardiovascular diseases. Deficiency of apoE results in massive accumulation of remnant lipoproteins, leading to severe hypercholesterolemia and atherosclerosis in human and apoE knockout mice [1]. Here, we reported that HC individuals have lower *APOE *mRNA expression than NL individuals, which is concordant with the previous information related to apoE deficiency.

The *APOE *mRNA expression is extremely complex with regulation in a tissue-specific manner and in response to cellular changes and extra and intra-cellular factors [1]. The expression of cholesterol acceptors in the efflux process such as apoAI, as well as transporter proteins involved in this process have been described to activate *APOE *transcription in human adipocytes and macrophages [9,10]. Accordingly, we observed that individuals with low expression of *APOE *present a decreased apoAI plasma concentration. Moreover, we also reported that these individuals have increased concentrations of total and LDL cholesterol and apoB. The relation of plasma levels of LDL cholesterol with *APOE *expression in PBMC was previously reported in children with obesity [11]. The increased concentrations of particles which depend on the LDL receptor (LDLR) for their clearance from plasma is consistent with the key role of apoE as a high affinity ligand for the LDLR in the cholesterol homeostasis [12].

*APOE *allele frequencies have demonstrated to be heterogeneous among different populations, but the ε3 allele is almost invariably the most common and ε2 the rarest allele [13]. In this study, the frequencies of *APOE *alleles observed in the overall population (ε2: 5%, ε3: 79% and ε4:16%) were similar to earlier studies in European descendant population [13,14], African American [15] and Brazilian populations [16].

Several genetic factors have been related to hypercholesterolemia, however in most of the cases the contribution of these genetic factors to the risk for hypercholesterolemia depends on environmental factors. Our results showed differences in genotype and allele frequencies between normolipidemic and hypercholesterolemic groups, suggesting that *APOE *ε2 allele confers protection against hypercholesterolemia. This characteristic persists even after adjustment for covariates that have been largely associated with hypercholestolemia, such as gender, ethnics, history of CAD, age, hypertension, obesity, cigarette smoking and physical activity, suggesting that *APOE *ε2 could be considered an independent factor that protect against hypercholesterolemia in our sample population.

Ferreira and co-workers [16] did not found differences in *APOE *ε2/ε3/ε4 genotypes between normolipidemic and dyslipidemic Brazilian individuals. On the other hand, and in line with our results, it has been reported higher frequency of ε2 allele in normolipidemic than hypercholesterolemic individuals from South America [17]. Moreover, a previous study in the Brazilian population reported that, compared with the ε2 allele, the presence of ε3 allele increases more than two times the risk for dyslipidemia (OR: 2.31, CI:1.06-5.06) [18], which is in agreement with our results.

The effects of *APOE *polymorphisms on plasma lipids have been described by several studies and the evidence suggests that *APOE *ε2 is associated with lower, whereas ε4 with higher, concentrations of plasma total cholesterol, LDL cholesterol and apoB in comparison with the ε3 allele [19]. We reported a less atherogenic lipid profile of ε2 allele, as well as a contribution of ε4 allele for higher total and LDL cholesterol and apoB in normolipidemic individuals. However, the association between *APOE *polymorphisms and plasma lipids were detected exclusively in the normolipidemic group. In agreement with this characteristic, an association of *APOE *genotypes with basal plasma lipids in normolipidemic individuals, but not in dyslipidemic patients, was previously reported in our population [16]. The authors described that ε2 allele carriers had significantly lower total, LDL and non-HDL cholesterol compared to ε3 and ε4 allele carriers only in normolipidemic individuals. Moreover, other studies were not able to demonstrate any association between *APOE *ε2/ε3/ε4 genotypes and total and LDL cholesterol in patients with familial hypercholestolemia [20] and polygenic dyslipidemia [21,22]. Nerveless, the lack of association of *APOE *polymorphisms with plasma lipids in hypercholesterolemic patients in our sample seems to be attributable to the small number of individuals carrying the ε2 allele that could be considered an important limitation of our study.

The variation on plasma lipids according to *APOE *ε2/ε3/ε4 genotypes are believed to stem mainly from structural and biophysical properties of apoE isoforms [3]. ApoE4-containing lipoproteins exhibit a high binding ability to their receptors that cause a more efficient catabolism and an accelerated clearance of chylomicrons and VLDL-remnants, leading to down regulation of LDLR and HMGCR and to increased LDL cholesterol levels in plasma. On the contrary, lipoproteins containing the apoE2 isoform present lower affinity compared to apoE4 and apoE3 isoforms that result in decreased cholesterol levels.

In the present study, no differences were observed in the change of lipid levels in response to atorvastatin treatment according to *APOE *genotypes. Although many studies have evaluated the influence of *APOE *polymorphism on statin response, some of these studies had controversial results. Whereas there is a strong line of evidence linking *APOE *ε2/ε3/ε4 genotypes with the efficacy of statin treatment [23-26], other studies did not reveal any association between *APOE *genotypes and response to treatments with various statins [27-29]. Commonly, evidence supports that *APOE *ε3 allele is associated with better response than ε4 allele in term of LDL cholesterol decrease and, in addition, individuals carrying the ε2 allele have greater reduction of LDL cholesterol than ε3 homozygotes [30]. These differences result from the improved activity of HMGCR in ε2 compared to ε3 allele carriers due to the modulation of intracellular cholesterol by the upregulation of hepatic LDLR, which has lower affinity for the apoE2 isoform that results in an improved response of ε2 allele carriers to the inhibition of HMGCR by statins. On the other hand, the LDLR presents higher affinity for apoE4 isoform and the effect of statin therapy is diminished in ε4 allele carriers when compared to ε2 or ε3 [3].

In the last years, GWAS have provided new perspectives and a more comprehensive approach for identifying genetic loci associated to statin response. Thompson *et al*. (2009), using a platform of 291, 988 SNPs did not observe any association between genotypes and atorvastatin response at beginning, when 1, 984 individuals were analyzed, however further analysis in 5745 individuals from the Treating to New Target (TNT) trial using a candidate gene approach reported a strong association between *APOE *ε2/ε3/ε4 genotypes and LDL cholesterol statin response [31]. Furthermore, a recent study has evaluated the response to diverse statins using a GWAS approach involving nearly 4, 000 individuals from three different trials of statin efficacy [Cholesterol and Pharmacogenetics (simvastatin), Pravastatin/Inflammation CRP evaluation (pravastatin) and TNT (atorvastatin)] [32]. The authors did not found any association between *APOE *SNPs and statin response, however the SNP rs4429638, located in the *APOC1 *gene and near *APOE*, was associated with change LDL cholesterol suggesting a possible involvement of *APOE *locus in statin efficacy.

Despite the number of studies investigating the response to statins according to *APOE *genotypes, the effect of HMGCR inhibitors on apoE protein and mRNA expression has been poorly studied, particularly using *in vivo *models. Atorvastatin and cerivastatin demonstrated to reduce apoE protein secretion and *APOE *mRNA expression in THP-1 derived macrophages after 24h of treatment in a dose dependent manner [33]. Conversely, in cultured human monocyte-derived macrophages, lovastatin increased *APOE *mRNA levels but decreased apoE secretion [34], phenomena that the authors attributed to the increase of apoE not destined for secretion. On the other hand, regarding *in vivo *studies, Guan *et al*. reported that *APOE *mRNA levels in mononuclear cells of hyperlipidemic diabetic patients taking simvastatin (5-10 mg/day) did not differ from those without statin treatment [35]. These contradictory results from *in vitro *experiments and the data reported for Guan and co-workers and our observations in PBMC from hypercholesterolemic individuals (not change after 10 mg/day atorvastatin treatment) could be explained by the differences in the cellular models used by the authors. Moreover, we observed that patients without LDL cholesterol goal achievement had lower *APOE *mRNA expression that could suggest a possible involvement of the modulation of this gene in the statin response.

## Conclusions

Hypercholesterolemic patients had lower *APOE *mRNA levels than normolipidemic individuals and the *APOE *expression levels were associated with differences in the plasma lipid profile, corroborating the key role of the *APOE *in the cholesterol metabolism and suggesting that *APOE *mRNA expression may be a good marker for hypercholesterolemia in our sample population. Moreover, although there was no effect of ε2/ε3/ε4 polymorphisms on *APOE *gene expression, the presence of ε2 allele confers protection against hypercholesterolemia in Brazilian subjects. Moreover, there was no evidence of an involvement of *APOE *genotypes with atorvastatin lipid-lowering response and the atorvastatin treatment does not modify the mRNA expression in hypercholesterolemic subjects, however the low *APOE *mRNA expression after treatment showed by individuals with poor cholesterol lowering-response suggests a possible involvement of *APOE *level in atorvastatin response.

## Methods

### Study population and therapeutic protocol

Characteristic of study population and therapeutic protocol were previously described [36]. Three-hundred-sixty-two (255 women and 107 men, aged 29 to 81 y) individuals were randomly selected at the University Hospital of University of Sao Paulo and the Institute Dante Pazzanese of Cardiology, Sao Paulo city, Brazil. One-hundred-eighty-one subjects were classified as hypercholesterolemic (HC) according to the IV Brazilian Guidelines for Dyslipidemia and Atherosclerosis Prevention [8] and 181 were considered normolipidemics (NL) LDL cholesterol < 3.36 mmol/L (130 mg/dL)]. Individuals with diabetes mellitus; hypertriglyceridemia [triglycerides > 4.42 mmol/L (350 mg/dL)]; liver, renal or thyroid disease; pregnant women or under treatment of oral contraceptives; and other causes of secondary dyslipidemia were not included in the study.

HC individuals went through a four-week washout having a low fat diet according to the American Heart Association recommendation [37]. One-hundred-forty-one individuals from HC group had indication of lowering-cholesterol drug therapy in order to reach the LDL cholesterol goal according to the IV Brazilian Guidelines for Dyslipidemia and Atherosclerosis Prevention [8]. Afterwards, these individuals were treated with 10 mg/day of atorvastatin during 4 weeks (ATORVA group). Serum lipids were measured to evaluate atorvastatin cholesterol-lowering response. The study protocol was approved by the ethics committees of the Institute Dante Pazzanese of Cardiology, University Hospital and the School of Pharmaceutical Sciences of the University of the Sao Paulo. Each individual agreed to participate in the study by signing an informed consent.

### Biochemical measurements

Blood samples were collected after an overnight (12 h) fast. ATORVA patients had blood drown previous and after the 4-week atorvastatin treatment. Plasma total cholesterol, HDL cholesterol and triglycerides were measured by routine enzymatic colorimetric methods. Plasma apo AI and apo B were measured by nephelometry. LDL and VLDL cholesterol were estimated by Friedewald formula [38]. Serum ALT and CK concentrations were determined by kinetic methods to evaluate atorvastatin effects on liver and muscle tissues.

### *APOE *genotyping

Genomic DNA was extracted from EDTA-treated blood samples using salting out procedure [39]. SNPs rs7412 and rs429358, that determinate the *APOE *ε2, ε3 and ε4 alleles were analyzed by allelic discrimination using TaqMan real time PCR system. Validated SNP genotyping assays (ID number C_904973_1 for rs429358 and C_904973_1 for rs7412) were purchased from Applied Biosystems (Applied Biosystems, CA, USA). Both SNP genotyping reactions were optimized in a total volume of 8 μl using 20 ng of DNA and fluorescence was detected in a 7500 Fast Real-Time PCR system (Applied Biosystems, CA, USA). Control samples with known *APOE *genotypes were included in each PCR run, which were analyzed by the alternative PCR-RFLP method [40].

### *APOE *mRNA quantification in PBMC

*APOE *mRNA expression was measured in individuals from NL (n = 88) and ATORVA (n = 94) groups. EDTA-anticoagulated blood samples were used to obtain peripheral blood mononuclear cells (PBMC) as previously described [41] and immediately used for RNA extraction. Total RNA was extracted from PMBC using TRIzol^® ^Reagent (Invitrogen-Life Technologies, CA, USA) following the manufacturer's suggested protocol. RNA was dissolved in DEPC-treated water and the concentration was measured by spectrophotometry using the NanoDrop^® ^(NanoDrop Technologies INC., DE, USA). RNA integrity was evaluated using the bioanalyzer^®^2100 (Agilent technologies, CA, USA). Samples with RNA integrity number (RIN) lower than 5 were not used for mRNA experiments. cDNA was produced from 1 μg of total RNA by Superscript™ II Reverse Transcriptase (Invitrogen-Life Technologies, CA, USA).

*APOE *mRNA expression was measured by quantitative TaqMan real-time PCR (qPCR). The assay ID Hs00171168_m1 was used to access the *APOE *mRNA detection. Genorm software http://medgen.ugent.be/genorm was used to select the most stable among six endogenous reference genes [ubiquitin C (*UBC*), glyceraldehyde-3-phosphate dehydrogenase (*GAPD*), beta-2-microglobulin (*B2M*), Hypoxanthine phosphoribosyl-transferase I (*HPRTI*), succinate dehydrogenase complex, subunit A (*SDHA*) and hydroxymethyl-bilane synthase (*HMBS*)], and the most stable in the experimental conditions was *UBC*. The sequence of primers and probes used for *UBC *are described allows: Forward, 5'-ATTTGGGTCGCGGTTCTTG-3'; reverse, 5'-TGCCTTGACATTCTCGAT GGT-3'; and probe, VIC -TCGTCACTTGACAATGC- MGB/NFQ. The qPCR assays were carried out in 96 well plates using a 7500 Fast Real-Time PCR system (Applied Biosystems, CA, USA). The relative quantification of *APOE *mRNA was calculated by the comparative Ct method using the formula 2^-ΔCt ^[42].

### Statistical Analysis

Statistical analyses were performed using SPSS v.15 for windows (SPSS Inc., Madrid, Spain) and Minitab v.15 statistical software (Minitab Inc. State College, PA). The allele frequencies and genotype distribution were estimated by gene counting. SNPanalyzer software v. 1.0 [43] was used to verify whether the genotypes distribution were in Hardy-Weinberg equilibrium (HWE) using the expectation-maximization algorithm. Differences in non-continuous variables, genotype and allelic distributions were compared by chi-square test. Normality distribution for all continuous variables was tested by Kolmogorov-Smirnov test and skewed variables were log transformed to improve normality for statistical analysis. Differences in mean values were evaluated by *t*-test or one-way ANOVA using Tukey post-hoc test. Paired *t*-test was used to analyze the effects of treatment in ATORVA group. In order to evaluate effects of *APOE *genotypes on the different variables, individuals were classified into three phenotypes: 1) E2 group, carrying the ε2ε2 and ε2ε3 genotypes; 2) E3 group, carrying the ε3ε3 genotypes; and 3) E4 group carrying either ε3ε4 or ε4ε4 genotypes. Individuals with the ε2ε4 were not assigned to any group and they were therefore excluded for the analysis. Mann Whitney U test or Kruskal-Wallis (two or three independent samples) or Wilcoxon signed rank test (pared samples) were used to evaluate differences in *APOE *mRNA gene expression. Correlations of *APOE *expression with other continuous variables were accessed by Spearman correlation test. Individuals were grouped in three groups (terciles) according to *APOE *expression values and plasma lipid concentrations were then compared among the groups using one-way ANOVA and Tukey post-hoc test. Multiple logistic regression analysis was used to examine the association of *APOE *genotypes with hypercholesterolemia including adjustment for relevant covariates. Multiple linear regression analysis was performed introducing variables of interest as dependent variables and *APOE *genotypes together to relevant covariates as independent variables, which regression coefficient were obtained for each independent variable. Statistical significance was set for p < 0.05.

## List of abbreviations

ApoE: apolipoprotein E; *APOE*: apolipoprotein E gene; HDL: high density lipoprotein; LDL: low density lipoprotein; VLDL: very low density lipoprotein; LDLR, low density lipoprotein receptor; HMGCR: 3-hydroxy-3-methylglutaryl coenzyme A reductase; HC: hypercholesterolemic; NL: normolipidemic; ATORVA: group of patients treated with atorvastatin; PBMC; peripheral blood mononuclear cells; CAD: coronary artery disease; GWAS: genome-wide association studies; SNP: single nucleotide polymorphism.

## Competing interests

The authors declare that they have no competing interests.

## Authors' contributions

AC carried out the experimental procedures, patients' selection, analysis of data and drafted the manuscript. FDVG, MAVW and SSA contribute to patients' selection and experimental procedures. MMSB, ELD, MCB and AAF contribute to patients' selection and monitoring patients under study protocol. MHH and RDCH participated in the design of the study, interpretation of data and elaboration of the manuscript. All authors have read and approved the final manuscript.

## Supplementary Material

Additional file 1 **Supplementary table**. Influence of 10 mg/day/4-weeks atorvastatin treatment on plasma lipids in ATORVA group (n = 141). Lipid plasma concentrations in individuals of the ATORVA group at baseline and after 10 mg/day/4-weeks atorvastatin treatment. Click here for file

## References

[B1] Greenow K Pearce NJ Ramji DP The key role of apolipoprotein E in atherosclerosis J Mol Med 2005 83 329 342 10.1007/s00109-004-0631-3 15827760

[B2] Strittmatter WJ Bova Hill C Molecular biology of apolipoprotein E Curr Opin Lipidol 2002 13 119 123 10.1097/00041433-200204000-00002 11891413

[B3] Huang Y Mechanisms linking apolipoprotein E isoforms with cardiovascular and neurological diseases Curr Opin Lipidol 2010 21 337 345 10.1097/MOL.0b013e32833af368 20531185

[B4] Baigent C Keech A Kearney PM Blackwell L Buck G Pollicino C Kirby A Sourjina T Peto R Collins R Simes R Efficacy and safety of cholesterol-lowering treatment: prospective meta-analysis of data from 90, 056 participants in 14 randomised trials of statins Lancet 2005 366 1267 1278 16214597 10.1016/S0140-6736(05)67394-1

[B5] Mangravite LM Thorn CF Krauss RM Clinical implications of pharmacogenomics of statin treatment Pharmacogenomics J 2006 6 360 374 10.1038/sj.tpj.6500384 16550210

[B6] Zintzaras E Kitsios GD Triposkiadis F Lau J Raman G APOE gene polymorphisms and response to statin therapy Pharmacogenomics J 2009 9 248 257 10.1038/tpj.2009.25 19529002

[B7] Hirata RDC Hirata MH Genome-wide and candidate genes approach for pharmacogenomics of atorvastatin Clinical Lipidology 2009 4 419 423 10.2217/clp.09.32

[B8] Sposito AC Caramelli B Fonseca FA Bertolami MC Afiune Neto A Souza AD Lottenberg AM Chacra AP Faludi AA Loures-Vale AA IV Brazilian Guideline for Dyslipidemia and Atherosclerosis prevention: Department of Atherosclerosis of Brazilian Society of Cardiology Arq Bras Cardiol 2007 88 Suppl 1 2 19 17515982 10.1590/s0066-782x2007000700002

[B9] Bencharif K Hoareau L Murumalla RK Tarnus E Tallet F Clerc RG Gardes C Cesari M Roche R Effect of apoA-I on cholesterol release and apoE secretion in human mature adipocytes Lipids Health Dis 2010 9 75 10.1186/1476-511X-9-75 20642861 PMC2917427

[B10] Von Eckardstein A Langer C Engel T Schaukal I Cignarella A Reinhardt J Lorkowski S Li Z Zhou X Cullen P Assmann G ATP binding cassette transporter ABCA1 modulates the secretion of apolipoprotein E from human monocyte-derived macrophages FASEB J 2001 15 1555 1561 10.1096/fj.00-0798com 11427487

[B11] Xiang W Ma YL Chen C Fu SM Yang JF Zhao SP Guo DX Zhao DC Nie S Wang FL Apolipoprotein E gene expression in peripheral blood monocyte in children with obesity Zhonghua Er Ke Za Zhi 2003 41 755 760 14731357

[B12] Pendse AA Arbones-Mainar JM Johnson LA Altenburg MK Maeda N Apolipoprotein E knock-out and knock-in mice: atherosclerosis, metabolic syndrome, and beyond J Lipid Res 2009 50 Suppl S178 182 19060252 10.1194/jlr.R800070-JLR200 PMC2674752

[B13] Schiele F De Bacquer D Vincent-Viry M Beisiegel U Ehnholm C Evans A Kafatos A Martins MC Sans S Sass C Apolipoprotein E serum concentration and polymorphism in six European countries: the ApoEurope Project Atherosclerosis 2000 152 475 488 10.1016/S0021-9150(99)00501-8 10998477

[B14] Sima A Iordan A Stancu C Apolipoprotein E polymorphism--a risk factor for metabolic syndrome Clin Chem Lab Med 2007 45 1149 1153 10.1515/CCLM.2007.258 17848120

[B15] Cavallari LH Langaee TY Momary KM Shapiro NL Nutescu EA Coty WA Viana MA Patel SR Johnson JA Genetic and clinical predictors of warfarin dose requirements in African Americans Clin Pharmacol Ther 2010 87 459 464 10.1038/clpt.2009.223 20072124

[B16] Ferreira CN Carvalho MG Fernandes AP Lima LM Loures-Valle AA Dantas J Janka Z Palotas A Sousa MO Comparative study of apolipoprotein-E polymorphism and plasma lipid levels in dyslipidemic and asymptomatic subjects, and their implication in cardio/cerebro-vascular disorders Neurochem Int 2010 56 177 182 10.1016/j.neuint.2009.09.016 19819279

[B17] Arraiz N Bermudez V Prieto C Sanchez MP Escalona C Sanz E Rondon N Reyes F Velasco M Association between apoliprotein E gene polymorphism and hypercholesterolemic phenotype in Maracaibo, Zulia state, Venezuela Am J Ther 2010 17 330 336 10.1097/MJT.0b013e3181c1235d 20019593

[B18] Mendes-Lana A Pena GG Freitas SN Lima AA Nicolato RL Nascimento-Neto RM Machado-Coelho GL Freitas RN Apolipoprotein E polymorphism in Brazilian dyslipidemic individuals: Ouro Preto study Braz J Med Biol Res 2007 40 49 56 10.1590/S0100-879X2007000100007 17224996

[B19] Gronroos P Raitakari OT Kahonen M Hutri-Kahonen N Marniemi J Viikari J Lehtimaki T Influence of apolipoprotein E polymorphism on serum lipid and lipoprotein changes: a 21-year follow-up study from childhood to adulthood. The Cardiovascular Risk in Young Finns Study Clin Chem Lab Med 2007 45 592 598 10.1515/CCLM.2007.116 17484618

[B20] Christidis DS Liberopoulos EN Kakafika AI Miltiadous GA Cariolou M Ganotakis ES Mikhailidis DP Elisaf MS The effect of apolipoprotein E polymorphism on the response to lipid-lowering treatment with atorvastatin or fenofibrate J Cardiovasc Pharmacol Ther 2006 11 211 221 10.1177/1074248406293732 17056835

[B21] Kathiresan S Willer CJ Peloso GM Demissie S Musunuru K Schadt EE Kaplan L Bennett D Li Y Tanaka T Common variants at 30 loci contribute to polygenic dyslipidemia Nat Genet 2009 41 56 65 10.1038/ng.291 19060906 PMC2881676

[B22] Willer CJ Sanna S Jackson AU Scuteri A Bonnycastle LL Clarke R Heath SC Timpson NJ Najjar SS Stringham HM Newly identified loci that influence lipid concentrations and risk of coronary artery disease Nature Genetics 2008 40 161 169 10.1038/ng.76 18193043 PMC5206900

[B23] Takane H Miyata M Burioka N Shigemasa C Shimizu E Otsubo K Ieiri I Pharmacogenetic determinants of variability in lipid-lowering response to pravastatin therapy J Hum Genet 2006 51 822 826 10.1007/s10038-006-0025-1 16917677

[B24] Marques-Vidal P Bongard V Ruidavets JB Fauvel J Perret B Ferrieres J Effect of apolipoprotein E alleles and angiotensin-converting enzyme insertion/deletion polymorphisms on lipid and lipoprotein markers in middle-aged men and in patients with stable angina pectoris or healed myocardial infarction Am J Cardiol 2003 92 1102 1105 10.1016/j.amjcard.2003.06.008 14583365

[B25] Thompson JF Man M Johnson KJ Wood LS Lira ME Lloyd DB Banerjee P Milos PM Myrand SP Paulauskis J An association study of 43 SNPs in 16 candidate genes with atorvastatin response Pharmacogenomics J 2005 5 352 358 10.1038/sj.tpj.6500328 16103896

[B26] Donnelly LA Palmer CN Whitley AL Lang CC Doney AS Morris AD Donnan PT Apolipoprotein E genotypes are associated with lipid-lowering responses to statin treatment in diabetes: a Go-DARTS study Pharmacogenet Genomics 2008 18 279 287 10.1097/FPC.0b013e3282f60aad 18334912

[B27] Vega GL Weiner M Kolsch H von Bergmann K Heun R Lutjohan D Nguyen A Moore C The effects of gender and CYP46 and apo E polymorphism on 24S-hydroxycholesterol levels in Alzheimer's patients treated with statins Curr Alzheimer Res 2004 1 71 77 10.2174/1567205043480546 15975088

[B28] Pena R Lahoz C Mostaza JM Jimenez J Subirats E Pinto X Taboada M Lopez-Pastor A Effect of apoE genotype on the hypolipidaemic response to pravastatin in an outpatient setting J Intern Med 2002 251 518 525 10.1046/j.1365-2796.2002.00991.x 12028507

[B29] Fiegenbaum M da Silveira FR Van der Sand CR Van der Sand LC Ferreira ME Pires RC Hutz MH Pharmacogenetic study of apolipoprotein E, cholesteryl ester transfer protein and hepatic lipase genes and simvastatin therapy in Brazilian subjects Clin Chim Acta 2005 362 182 188 10.1016/j.cccn.2005.06.005 16038892

[B30] Nieminen T Kahonen M Viiri LE Gronroos P Lehtimaki T Pharmacogenetics of apolipoprotein E gene during lipid-lowering therapy: lipid levels and prevention of coronary heart disease Pharmacogenomics 2008 9 1475 1486 10.2217/14622416.9.10.1475 18855536

[B31] Thompson JF Hyde CL Wood LS Paciga SA Hinds DA Cox DR Hovingh GK Kastelein JJ Comprehensive whole-genome and candidate gene analysis for response to statin therapy in the Treating to New Targets (TNT) cohort Circ Cardiovasc Genet 2009 2 173 181 10.1161/CIRCGENETICS.108.818062 20031582

[B32] Barber MJ Mangravite LM Hyde CL Chasman DI Smith JD McCarty CA Li X Wilke RA Rieder MJ Williams PT Genome-wide association of lipid-lowering response to statins in combined study populations PLoS One 2010 5 e9763 10.1371/journal.pone.0009763 20339536 PMC2842298

[B33] Castilho LN Chamberland A Boulet L Davignon J Cohn JS Bernier L Effect of atorvastatin on ApoE and ApoC-I synthesis and secretion by THP-1 macrophages J Cardiovasc Pharmacol 2003 42 251 257 10.1097/00005344-200308000-00015 12883330

[B34] Cignarella A Brennhausen B von Eckardstein A Assmann G Cullen P Differential effects of lovastatin on the trafficking of endogenous and lipoprotein-derived cholesterol in human monocyte-derived macrophages Arterioscler Thromb Vasc Biol 1998 18 1322 1329 10.1161/01.ATV.18.8.1322 9714140

[B35] Guan JZ Tamasawa N Murakami H Matsui J Tanabe J Matsuki K Yamashita M Suda T HMG-CoA reductase inhibitor, simvastatin improves reverse cholesterol transport in type 2 diabetic patients with hyperlipidemia J Atheroscler Thromb 2008 15 20 25 10.5551/jat.E512 18270459

[B36] Genvigir FD Soares SA Hirata MH Willrich MA Arazi SS Rebecchi IM Oliveira R Bernik MM Dorea EL Bertolami MC Hirata RD Effects of ABCA1 SNPs, including the C-105T novel variant, on serum lipids of Brazilian individuals Clin Chim Acta 2008 389 79 86 10.1016/j.cca.2007.11.029 18164264

[B37] Chahoud G Aude YW Mehta JL Dietary recommendations in the prevention and treatment of coronary heart disease: do we have the ideal diet yet? Am J Cardiol 2004 94 1260 1267 10.1016/j.amjcard.2004.07.109 15541241

[B38] Friedewald WT Levy RI Fredrickson DS Estimation of the concentration of low-density lipoprotein cholesterol in plasma, without use of the preparative ultracentrifuge Clin Chem 1972 18 499 502 4337382

[B39] Cerda A Genvigir FD Arazi SS Hirata MH Dorea EL Bernik MM Bertolami MC Faludi AA Hirata RD Influence of SCARB1 polymorphisms on serum lipids of hypercholesterolemic individuals treated with atorvastatin Clin Chim Acta 2010 411 631 637 10.1016/j.cca.2010.01.002 20064494

[B40] Salazar LA Hirata MH Giannini SD Forti N Diament J Lima TM Hirata RD Seven DNA polymorphisms at the candidate genes of atherosclerosis in Brazilian women with angiographically documented coronary artery disease Clin Chim Acta 2000 300 139 149 10.1016/S0009-8981(00)00308-9 10958870

[B41] Cerda A Genvigir FD Rodrigues AC Willrich MA Dorea EL Bernik MM Arazi SS Oliveira R Hirata MH Hirata RD Influence of Polymorphisms and Cholesterol-Lowering Treatment on SCARB1 mRNA Expression J Atheroscler Thromb 2011 18 640 651 10.5551/jat.6544 21512283

[B42] Livak KJ Schmittgen TD Analysis of relative gene expression data using real-time quantitative PCR and the 2(-Delta Delta C(T)) Method Methods 2001 25 402 408 10.1006/meth.2001.1262 11846609

[B43] Yoo J Seo B Kim Y SNPAnalyzer: a web-based integrated workbench for single-nucleotide polymorphism analysis Nucleic Acids Res 2005 33 W483 488 10.1093/nar/gki428 15980517 PMC1160189

