# Supplementary material for: Apolipoprotein E mRNA expression in mononuclear cells from normolipidemic and hypercholesterolemic individuals treated with atorvastatin
Source: Lipids Health Dis. 2011 Nov 10;10:206. doi: 10.1186/1476-511X-10-206 (PMC3247903; doi:10.1186/1476-511X-10-206)
Supplement: Additional file 1 — Supplementary table. Influence of 10 mg/day/4-weeks atorvastatin treatment on plasma lipids in ATORVA group (n = 141). Lipid plasma concentrations in individuals of the ATORVA group at baseline and after 10 mg/day/4-weeks atorvastatin treatment. [file 1476-511X-10-206-S1.DOC]

**Additional File 1**

**Supplementary table**. Influence of 10 mg/day/4-weeks atorvastatin treatment on serum lipids in ATORVA group (n=141)

| **Parameter** | **Baseline** | **Treatment** | **Change (%)** | **p-value** |
| --- | --- | --- | --- | --- |
| Total cholesterol (mg/dL) | 281±37 | 198±30 | -29±9 | <0.001 |
| LDL cholesterol (mg/dL) | 193±34 | 117±27 | -39±12 | <0.001 |
| HDL cholesterol (mg/dL) | 57±14 | 55±13 | -3±10 | <0.001 |
| VLDL cholesterol (mg/dL) | 31±13 | 26±11 | -13±29 | <0.001 |
| Triglycerides (mg/dL) | 157±65 | 130±53 | -13±29 | <0.001 |
| ApoAI (mg/dL) | 136±26 | 138±28 | +2±12 | 0.113 |
| ApoB (mg/dL) | 143±27 | 98±20 | -30± 12 | <0.001 |

- / + symbols indicate reduction and increment of each parameter. Values are presented as media±SD and compared by pared t test. LDL, low density lipoprotein; HDL, high density lipoprotein; VLDL, very low density lipoprotein. ApoAI, apolipoprotein AI; ApoB, apolipoprotein B. Conversion factors to convert to Systeme Internacional (SI) units are 0.02586 for cholesterol (mmol/l), 0.01129 for triglycerides (mmol/l) and 0.01 for apolipoproteins (g/l).
